# Cost of treating severe malaria in children in Africa: a systematic literature review

**DOI:** 10.1186/s12936-024-05173-w

**Published:** 2024-11-09

**Authors:** Amani Thomas Mori, Grace Mallange, Melf-Jakob Kühl, Lucy Okell

**Affiliations:** 1 Section for Ethics and Health Economics, Department of Global Public Health and Primary Care, University of Bergen, P.O. Box 7804, 5020 Bergen, Norway; 2 Muhimbili University of Health and Allied Sciences, P.O. Box 65001, Dar es Salaam, Tanzania; 3 MRC Centre for Global Infectious Disease Analysis, Department of Infectious Disease Epidemiology, Imperial College, London, W2 1PG UK

**Keywords:** Severe malaria, Severe malarial anaemia, Child, Economic burden, Cost, Cost of illness, Africa

## Abstract

**Background:**

Malaria is a major cause of ill health and death in children in Africa. The disease also imposes a severe social and economic burden on households and health systems and is strongly associated with poverty. This study summarizes the most up-to-date cost of treating severe malaria in children in Africa.

**Methods:**

A systematic search was conducted in PubMed, Embase, Cinahl, and Web of Science databases. Google and Google Scholar were searched for grey literature followed by scanning of the reference lists of the previous systematic reviews. The search was limited to children < 12 years, malaria-endemic countries in Africa, and the English language. All costs were adjusted to the year 2023.

**Results:**

19 studies conducted in 12 countries were identified: 14 reported provider costs, and 11 household costs. Out of the 19 studies found, 11 were published before 2018 while 11 reported data that are currently more than ten years old. Studies varied methodologically and in the scope of resources included to estimate the cost. The provider costs ranged from USD 27 in Uganda to USD 165 per patient in Kenya (median value USD 90), while household costs ranged from USD 13 in Kenya to USD 245 per patient in Gabon (median value USD 50). All identified household malaria treatment costs except one represented catastrophic health expenditure, making out more than 10% of the monthly Gross National Income per capita in the respective countries.

**Conclusion:**

Evidence on the cost of treating severe malaria in children in Africa is scarce. However, the few existing studies show that severe malaria in children imposes a significant economic burden on the providers and households. More studies are needed, particularly in high-burden high-impact countries, to inform resource allocation decisions.

**Supplementary Information:**

The online version contains supplementary material available at 10.1186/s12936-024-05173-w.

## Background

Malaria is a major cause of disease burden in Africa, particularly in young children. The recent World Malaria Report estimated that in 2022, malaria caused 249 million cases and 608,000 deaths globally [1]. About 95% of these deaths occurred in Africa, and about 80% of them were in children younger than five years. About 70% of the malaria burden is concentrated in just 11 countries, of which ten are in Africa (Burkina Faso, Cameroon, DR Congo, Ghana, Mali, Mozambique, Niger, Nigeria, Uganda, and Tanzania) [1, 2]. Four of these countries, i.e., Nigeria, DR Congo, Niger, and Tanzania, were responsible for just over half of all malaria deaths [1]. In 2022, the total expenditure on malaria control and elimination globally was estimated at USD 4.1 billion, of which 80% went to Africa [1].

Malaria imposes a severe social and economic burden on households and health systems and is strongly associated with poverty [3]. In 2009, the financial cost of treating an episode of uncomplicated malaria and severe malaria was estimated at USD 5.84 (range 2.36–23.65) and USD 30.26 (range 15.64–137.87), respectively [4]. At the country level, the annual economic burden of malaria in 2020 ranged from USD 80 million in Ghana to USD 302 million in Kenya and USD 350 million in Tanzania [5]. In Zambia, treatment of severe malaria in children accounted for 7.7% of monthly household income [6]. In India, Tanzania, and Kenya, between 3% and 6.5% of household income or consumption expenditure were estimated to be committed to malaria treatment [7–9]. Out-of-pocket payment for malaria treatment exposes households to catastrophic health expenditure (CHE) when they exceed 10% of household income/total household consumption or 40% of non-food expenditure [10, 11]. In DR Congo, the incidence of CHE due to severe malaria in children was 81% for the 40% threshold and 46% for the 10% of total household consumption threshold [12]. In Sudan, South Africa, Mozambique and Zimbabwe the incidence of CHE due to malaria was estimated to vary between 17 and 32% [13–15].

Despite the known economic and social impact of malaria in Africa, the evidence on the unit costs of severe malaria treatment in children is limited. A review from 2003 only found one study from Africa [16]. Another review in 2011 found three studies [4]. The third review from 2019 identified five studies [17]. The fourth review from 2021 identified six studies [18]. The most recent review from 2022 (last search in May 2020) identified seven studies [19]. This study aims to provide the most up-to-date health system and household unit costs for the treatment of severe malaria in children less than 12 years old to inform policy decisions and economic evaluation of post-discharge malaria chemoprevention (PDMC) [20]. PDMC is a new intervention recommended by the World Health Organization (WHO) against severe malarial anaemia in children in endemic countries in Africa [21]. This study focuses on this age group because the burden of severe malaria has been shifting towards older children due to reduced transmission [22–24].

## Methods

### Study design

This is a systematic review study and followed the Preferred Reporting Items for Systematic Reviews and Meta-Analysis (PRISMA) statement, with slight modifications to suit the review of costing studies [25]. The review was Registered in Prospero with Reg. No: CRD42023401799 [26].

### Search strategy and selection criteria

Four databases including PubMed, Embase, Cinahl and Web of Science were systematically searched. Google and Google Scholar were searched for grey literature followed by scanning of the reference lists of previous systematic reviews. The search had no date restrictions, and the last search was conducted on January 04, 2024. All studies, which involved children below 12 years old were included. However, studies with a small proportion of children exceeding this age were also included to ensure consistency with a previous systematic review that quantified the post-discharge risk of mortality and morbidity among children admitted with severe malaria and other syndromes [27]. Other inclusion criteria were that studies were conducted in malaria-endemic countries in Africa, they focused on the management of severe malaria (i.e., malaria requiring hospitalization) and reported primary cost data. Studies that exclusively relied on secondary data and studies on travelers from non-endemic countries were excluded.

An example of the search terms used in PubMed were: (severe malaria or cerebral malaria or severe malaria anaemia or severe malaria anemia or hospital malaria or inpatient malaria).ti,ab,kf. AND (((cost benefit or cost–benefit or cost effectiveness or cost-effectiveness or cost utility or cost analy* or cost and benefit or economic evaluation* or cost or economic benefit*).ti,ab,kf. AND ("Burkina Faso" or Burkinabe or Cameroon* or "Democratic Republic of the Congo" or Congolese or Ghana* or Mali* or Mozambique or Mozambican or Niger* or Tanzania* or Uganda* or Angola* or Benin or Beninese or Botswana or Motswana or Burundi* or "Central African Republic" or Chad* or Comoros or Comorian* or "Cote d'Ivoire" or "Ivory Coast*" or Djibouti* or "Equatorial Guinea*" or Eritrea* or Ethiopia* or Gabon* or Gambia* or Guinea* or Guinea-Bissau or Bissau-Guinean* or Kenya* or Liberia* or Madagascar or Malagasy or Malawi* or Maurit* or Namibia* or Rwand* or Senegal* or "Sierra Leone" or Somalia* or "South Africa*" or "South African" or "South Sudan*" or Sudan* or Togo* or Zambia* or Zimbabwe*).ti,ab,kf. An additional file shows all the search terms used for each database (see Additional file 1**).**

**Fig. 1:**
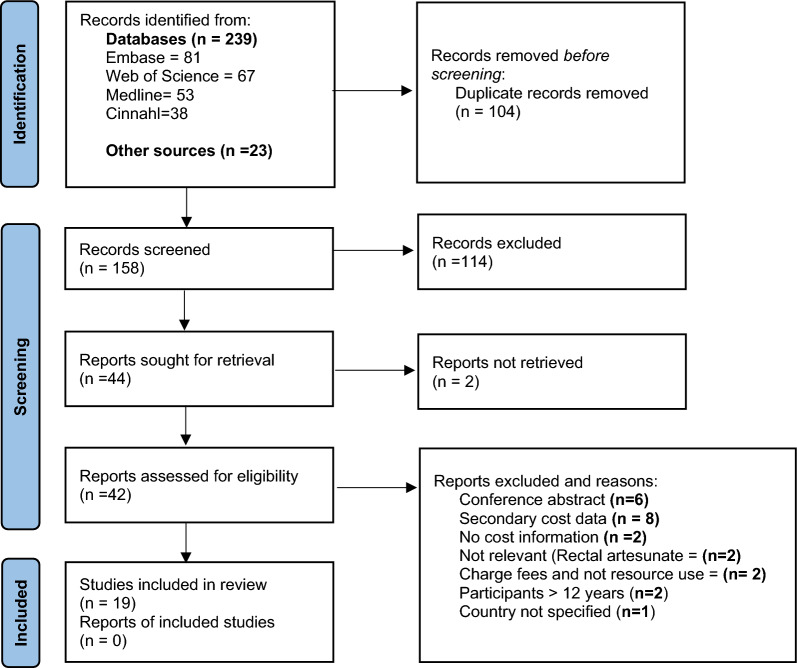
PRISMA flow diagram

Figure 1 is the PRISMA flow diagram. All identified articles and reports were screened by two researchers, GM and ATM independently and assessed for eligibility. Any disagreement was resolved by consensus, or by a third reviewer.

### Data extraction

The following information was extracted from each article when available: authors’ names, the year when the article was published and data were collected, the name of the country where cost data was collected, the currency used, costing perspective used, level of health care facility where costing was done, type of severe malaria, and patient age. Provider costs were those borne by the health facilities and household costs were borne by the patients’ caregivers. The societal costing perspective included both the provider and household costs. Provider costs were classified as capital (building space, equipment, vehicles, furniture) and recurrent costs (human resources, utilities, medicines, supplies/consumables, and training) [28], while household costs included direct medical costs (consultation, diagnosis, and medicines), direct non-medical costs (transport, food) and indirect costs (loss in productivity due to time used to seek care and to take care of a sick child at home) [29].

### Quality assessment

Quality assessment was conducted independently by ATM and GM. An 8-item checklist was developed from Drummond et al. [30], Liers et al. [31], the Consolidated Health Economic Evaluation Reporting Standards (CHEERS) [32] and the consensus-based checklist for the critical appraisal of cost-of-illness (COI) studies [33]. The included items were: (i) description of the characteristics of the study population and the reasons why it was chosen; (ii) the costing methodology used e.g. bottom-up, top-down, or a combination; (iii) the sources of resource utilization data (e.g. clinical trials, administrative databases, clinical databases, medical records, or published literature); (iv) resource quantities should be reported or described independently from the costs so that assessment of the measurement method is facilitated; (v) the viewpoint/perspective of the analysis such as the provider, patient and family, or societal perspectives should be clearly described; (vi) all costs should be adjusted to a specific price year to exclude inflation effects; vii) if the time horizon for estimating costs was longer than one year, discounting should have been performed to reflect time preferences; (viii) if prices were used instead of costs, they should reflect the true opportunity costs. Quality was assessed by scoring each item with a value of ‘1’ if fully completed, ‘0.5’ if not fully completed, ‘0’ if not completed and ‘NA’ if not applicable. The quality scores were categorized as ‘low’ if ≤ 33%, ‘moderate’ if the score was between 34 and 66% and ‘high’ if > 66%.

### Data analysis

All costs were converted to USD and then adjusted for Purchase Power Parity 2023 (PPP-USD) using the online cost converter tool by Campbell & Cochrane Economics Methods Group (CCEMG)—Evidence for Policy and Practice Information and Coordinating Centre (EPPI-Centre) [34]. Monthly gross national income (GNI) per capita [35] was used as a proxy of per capita household income. Household payments that exceeded 10% of this income were assumed to constitute Catastrophic Health Expenditure (CHE)[11].

### Assessment of heterogeneity

Although costing studies are inherently heterogeneous, partly because of the lack of standardized guidelines [33], the degree of heterogeneity of the included studies was assessed to decide whether to conduct a narrative synthesis or quantitative analysis [36]. Heterogeneity was assessed by examining the costing approach used, categorizes and types of costs included, setting e.g. urban versus rural, level (primary healthcare, hospitals) and ownership of the health facility (public, private, faith-based) for the provider perspective, age group and clinical condition e.g. severe malaria anaemia vs severe malaria.

## Results

### Study characteristics

19 studies conducted in 12 countries were identified. Eleven of these studies were used in the previous systematic reviews [37–47], and seven were newly included [48–55]. Twelve studies were conducted in children younger than five years old, while others included older children. Most of the studies were conducted in public or mission hospitals, most at district-level hospitals, and a few at regional or referral hospitals. A few studies were conducted in private hospitals. Twelve studies were published before 2019. Twelve studies were cross-sectional, six were conducted alongside or following clinical trials [41, 42, 46, 48, 49, 51], and one was an implementation study [39] (Table 1). All studies were of moderate to high quality.

**Table 1:** Study characteristics

Author and year	Country	Site setting	Level: Ownership	Age group
Kühl (2022) [51]	Malawi	Urban and rural: Zomba district	District hospital: public	6–59 months
Batura (2021) [48]	Uganda	Rural & peri urban: Midwestern	Hospitals: public and private	< 5 years
	Mozambique	Rural & peri urban: Inhambane province	Hospital: public	< 5 years
Moukoumbi (2021) [54]	Gabon	*Not described*	University hospital	< 5 years
Mbalabu (2021) [53]	DR Congo	Mbujimayi city-Eastern Kasai	Provincial hospital: public	5–59 months
Watts (2021) [52]	Kenya	Rural: Kakamega County	Sub-county hospital: public	< 5, 5–12, > 12 years
Alonso (2019) [42]	Mozambique	Rural: Mopeia district, central	District hospital: public	6 m-5 years
Hennessee (2017) [43]	Malawi	Urban and rural: Nationwide	Public and mission hospitals	< 5 years and above
Maka (2016) [46]	Cameroon	Rural & peri urban: Ebolowa region	Regional hospital: public	3 months-15 years
Ferrari (2015) [39]	DR Congo	Urban and rural: Masina, Kimpese, Kisantu and Maluku zones	Hospitals and health centers: public and private	2 months and above
Comfort (2014) [38]	Zambia	Rural: Choma district	Mission district hospital: public	< 6 years
		Urban & semi-urban:Livingstone district	Regional hospital: public	< 5 years
Mori (2014) [47]	Tanzania	Urban: Mwananyamala	District hospital: public	< 5 years
Ilunga-Ilunga (2014) [44]	DR Congo	Urban and Rural: Kinshasa	Public, private, confessional	< 5 and ≥ 5 years
Onwujekwe (2013) [40]	Nigeria	Urban: Enugu state	Secondary hospital: public: mission	< 5 years
Abotsi (2012) [45]	Ghana	Urban and rural: Upper East region	District and regional hospital: public	Infants
Lubell (2011) [41]	Tanzania	Rural: Muheza district	District hospital: public	< 15 years
		Rural: Korogwe district	District hospital: public	
	Uganda	Urban and rural: Mbarara	Tertiary hospital: public	
	Nigeria	Rural: Ilorin	Tertiary hospital: public	
Kone (2010) [50]	Burkina Faso	Rural: Nouna hospital	District hospital-public	< 5 years*
Ayieko (2009) [37]	Kenya	Urban and rural: Nairobi, Rift Valley, Nyanza	National, provincial, district: public and mission	< 5 years
Kirigia (1998) [49]	Kenya	Kilifi and Malindi areas	District & sub district hospitals-public	< 5 years
Kodhiambo (2020) [55]	Kenya	Rural: Homabay county	Dispensary to district hospitals-Public	< 12 years

^*^Did not specify age group; it was probably < 5 years based on another study that the authors compared their results with

### Distribution

**Fig. 2:**
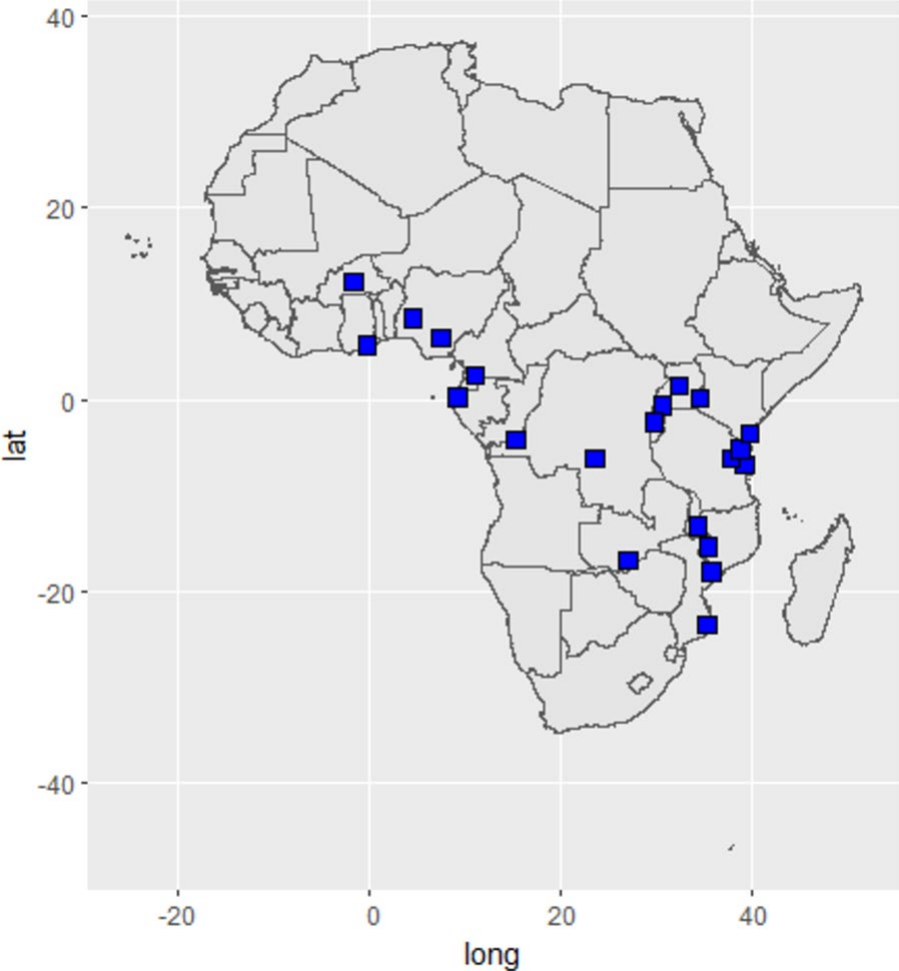
Map showing the study sites

Two included studies were conducted in more than one country [41, 48]. Twelve studies were conducted in East Africa (Tanzania, Kenya, Uganda and DR Congo) [37, 39, 41, 44, 47, 49, 52, 53, 55], five in Southern Africa (Malawi, Mozambique and Zambia) [38, 42, 48, 51] and five in West Africa (Nigeria, Ghana, Cameroon, Gabon and Burkina Faso) [40, 46, 50, 54] (Fig. 2). These countries represent eight of the ten African countries carrying the largest burden of malaria, the High Burden High Impact countries, which are high priority in global malaria control efforts [56].

### Provider costs of malaria treatment

Provider costs per case of severe malaria treated, as converted to 2023, ranged from USD 27 in Uganda to USD 165 in Kenya across the 14 studies that reported provider costs, with a median value of USD 90 (Table 2). The identified studies used different costing methods. The bottom-up/ingredients approach was most used, while two studies applied a mix of bottom-up and top-down approaches or activity-based costing (ABC) [48, 50], and one used time-driven activity-based costing (TDABC) [39]. Eight studies included both the capital and recurrent costs, while six included recurrent costs only [38, 39, 42, 45, 46, 51]. The capital and recurrent cost items included, the number of admission days, and the type of treatment used varied between the studies. Some studies reported unit costs for different types of antimalarials used, e.g. IV quinine versus artesunate [39, 41, 46] (in the table the costs of artesunate were reported because it is the recommended treatment for severe malaria and quinine is only used when artesunate is not available). Several studies did not report the type of malaria treatment used [37, 40, 45, 47, 48], or the number of admission days [40, 41, 46–48]. The study by Batura et al*.* [48], which reported the lowest cost estimates, used cost data that were collected between 2013 and 2014 in Uganda and combined top-down and bottom-up approaches. The study by Kirigia et al. [49], reported the highest cost for severe malarial anaemia in Kenya, using data collected in 1993.

**Table 2:** Summary of provider costs (USD)

Author	Country	Base year	Cost category	Condition	Admission days	Base year cost	Approach	Cost (2023) (USD)
Kühl [51]	Malawi	2018	Recurrent only	SMA^a^	4.6	90.96	Ingredients	108.74
Batura [48]	Uganda	2021	Capital and recurrent	SM	–	24.4	Mixed	27.07
	Mozambique	2021	Capital and recurrent	SM	–	52.3	Mixed	58.02
Watts [52]	Kenya	2020	Capital and recurrent	SM	3	42.0	Ingredients	46.59
Alonso [42]	Mozambique	2017–2018	Recurrent	SM	5	36.97	Ingredient	44.19
Maka [46]	Cameroon	2016	Recurrent	SM	1.97 (0–4)	65.14	Ingredients	81.26
Ferrari [39]	DR. Congo	2013	Recurrent	SM	2 (1–9)	51.94	TDABC	67.33
Comfort [38]	Zambia*	2008	Recurrent	SM	9.3	64.7	Ingredients	90.39
		2008	Recurrent	SMA		85.78	Ingredients	119.83
		2008	Recurrent	CM		41.84	Ingredients	58.45
	Zambia**	2008	Recurrent	SM	7	62.52	Ingredients	87.34
		2008	Recurrent	SMA		63.41	Ingredients	88.58
		2008	Recurrent	CM		62.39	Ingredients	87.16
Mori [47]	Tanzania	2012	Capital and recurrent	SM	–	76.46	Ingredients	100.85
Onwujekwe [40]	Nigeria	2011	Capital and recurrent	SM	–	48.02 + 30.5^b^	Ingredients	105.51
Abotsi [45]	Ghana	2007	Recurrent	SM	–	74.32	Ingredients	105.82
Lubel [41]	Tanzania^§^	2009	Capital and recurrent	SM	5	66.7	Ingredients	92.59
	Tanzania^§§^	2009	Capital and recurrent	SM		54.0	Ingredients	74.96
	Uganda	2009	Capital and recurrent	SM		59.6	Ingredients	82.73
	Nigeria	2009	Capital and recurrent	SM	–	108.7	Ingredients	150.89
Kone [50]	Burkina Faso	2005	Capital and recurrent	SMA	3.5	61.08	Mixed	92.07
			Capital and recurrent	SMN	7.5	74.29	Mixed	111.98
Ayieko [37]	Kenya^†^	2005	Capital and recurrent	SM	5	95.58	Ingredients	144.08
	Kenya^††^	2005	Capital and recurrent	SM	–	62.42	Ingredients	94.09
Kirigia [49]	Kenya^¥^	1993/94	Capital and recurrent	SM	–	57.0	Ingredients	106.87
		1993/94	Capital and recurrent	SMA	–	87.8	Ingredients	164.62
	Kenya^¥¥^	1993/94	Capital and recurrent	SM	–	34.5	Ingredients	64.69
		1993/94	Capital and recurrent	SMA	–	40.4	Ingredients	75.75

Base year is the year when the cost data in the included studies were collected

^a^They mixed severe anaemia and severe malaria anaemia

^b^Capital costs were expressed per patient per year, and then adjusted for an overall mean of 6 days of hospitalization per admission

^*^Macha hospital, ^**^Livingstone hospital, ^§^Teule hospital, ^§§^Korogwe hospital, ^†^National level ^††^District, SM-severe malaria, ^¥^Kilifi ^¥¥^Malindi, SMA-severe malaria anaemia, SMN-severe malaria with neurological sequelae, TDABC-Time Driven Activity Based Costing

### Household costs for malaria treatment

All studies collected household data through interviews with parents or caregivers of the admitted children except one that used medical records to identify direct costs. Interviews were either based on household surveys or study exit interviews. There was a lot of variation in cost items included. For example, some studies included both direct medical (consultation fees, diagnosis, medicines) and non-medical costs (transport, food, drinks) and indirect costs; others included only one or two of these categories. Household costs associated with the treatment of severe malaria in children ranged from USD 13 in Kenya to USD 245 per patient in Gabon, with a median value of USD 50 (Table 3). The study by Moukoumbi et al*.* [54] in Gabon was conducted in a university hospital, with the country’s largest and best-equipped paediatric unit. Ilunga-Ilunga et al. in DR Congo included direct medical and non-medical costs, indirect costs and costs that the caregivers incurred for self-medication, and visits to traditional healers, churches and health centers before their children were admitted to the study facilities[44]. Likewise, Alonso et al. [42] in Mozambique included costs of traditional healers and self-treatment.

**Table 3:** Summary of household costs (USD)

Authors name	Country	Base year	Cost category	Condition	Base year cost	Method	Cost (2023) (USD)
Kühl [51]	Malawi	2018	Direct and indirect costs	SM	12.06	Interviews	14.42
	Malawi	2018	Direct and indirect costs	SMA	45.88	Interviews	54.85
Moukoumbi [54]	Gabon	2018–2019	Direct medical costs	SM	186 Euros	Review of records	244.64
Mbalabu [53]	DR Congo	2013	Direct medical costs	SM	38.6	Interviews	50.04
Watts [52]	Kenya	2020	Medical, non-medical and indirect costs	SM	16.4	Interviews	19.01
Alonso [42]	Mozambique	2017–2018	Prehospital costs, Medical, transportation, indirect costs	SMA	65.91	Interviews	78.79
Hennessee [43]	Malawi	2012	Medical, non-medical and indirect costs	SM	9.74	Interviews	12.85
Ilunga-Ilunga [44]	DR Congo	2011	Prehospital costs, Direct medical, non-medical and indirect costs	SM	179	Interviews	232.46
Onwujekwe [40]	Nigeria	2011	Medical cost, transport &indirect costs	SM	23.2	Interviews	31.17
Abotsi [45]	Ghana	2007	Transport, indirect cost	SM	46.62	Interviews	66.38
Ayieko [37]	Kenya	2005	Transportation, indirect and other Out of Pocket payments	SM	16.12	Medical records and interviews with care providers	24.30
Kodhiambo [55]	Kenya	2016	Transportation, food, indirect and direct costs	SM	10.04	Interviews with caregiver	12.52

### Household malaria cost and catastrophic health expenditure

**Fig. 3:**
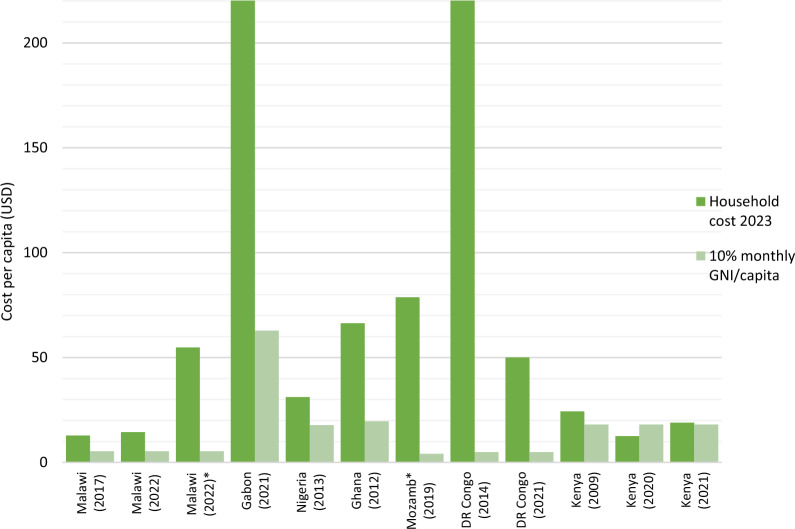
Household costs in 2023 versus 10% of per capita income. ^*^Severe malaria anaemia

As shown in Fig. 3, in all included studies except one[55], the household costs associated with severe malaria treatment in children represent catastrophic health expenditure, exceeding 10% of the monthly GNI per capita. Household costs were more than twice this threshold except for Kenya[37, 52] and Nigeria[40]. In Mozambique, household costs for treating severe malaria anaemia were 17 times higher than the income threshold[42], while in DR Congo, the cost for severe malaria treatment was 43 times higher than the income threshold[44].

## Discussion

This study updated the previous systematic review study by El-Houderi et al. [17] about the treatment costs of severe malaria in children in Africa. 19 studies reporting treatment costs of severe malaria in children in Africa were identified compared to five that were reported by the previous study. The large difference in the number of studies could be because of the inclusion of older children while the earlier study’s age limit was set at five years. The relatively low number of studies found in this review is surprising considering that the burden of malaria is concentrated in children. Twelve studies were conducted in eight of the ten High Burden High Impact countries identified by WHO: Uganda (2), Nigeria (2), Mozambique (2), Burkina Faso (1), Cameroon (1), Tanzania (1) and DR. Congo (3) [2].

Twelve of the identified studies were conducted more than five years ago, of which eleven used more than ten years old data. To inform prioritization decisions for malaria interventions, particularly the cost-effectiveness of preventive strategies, it is essential that the evidence on costing remains updated. Hence, new studies are needed that can accommodate the changing treatment protocols, coupled with the progress made in malaria control and health system strengthening in the past few years in malaria-endemic countries.

In the past two years, the WHO recommended the use of RTS,S malaria vaccine [56], and post-discharge malaria chemoprevention (PDMC) in children in Africa [20], both of which could have a significant impact on the prevalence and unit cost of managing severe malaria.

It is hard to make meaningful comparisons of treatment costs between studies conducted within the same country or different countries, regions, or based on different malaria transmission intensities because of the large variation in methodological approaches and costs included. Some studies used the bottom-up or ingredients approach while a few mixed top-down and ingredients or Activity-Based Costing (ABC) approaches [48, 50] and one used Time-Driven Activity-Based Costing (TDABC) approach [39]. The top-down approach apportions accumulated expenditure at a costing center down to units of activities or products while the bottom-up approach identifies, quantifies and values resources used for individual patients to obtain the total costs per case treated [57, 58]. On the contrary, ABC approaches break down the service into specific activities and identify the associated resources and their values (costs) [58]. In TDABC, time estimates resource consumption for each activity performed to deliver the service [59, 60]. The top-down approach is faster and able to capture inefficiency, but it is not as precise as bottom-up-informed costing[58]. On the contrary, a bottom-up method may underreport inefficiency and require adequate documentation to produce detailed cost information, which is particularly difficult in low-income countries [58]. Therefore, these approaches often produce different cost estimates [58, 61].

The provider cost items varied between studies, even those using the same costing approaches. Some studies included both capital and recurrent costs while others included only some recurrent costs [38, 39, 42, 45, 46, 51]. The type and range of included capital or recurrent cost items also varied. For example, the study by Maka et al. [46] did not include fixed or overhead costs because the authors compared two treatment options. The study by Moukoumbi et al. in Gabon was designed to estimate the provider costs, but it essentially only estimated the costs paid by the patients. It excluded the costs of managing the health facility, salaries, and wages of health care workers, while including hospitalization costs such as bed occupancy, food and beverages consumed and paid for by the patients [54].

The study designs also varied. Twelve studies were cross-sectional. However, six were conducted alongside randomized controlled trials or in trial sites, which may not reflect the routine practice [41, 42, 46, 48, 49, 51], and one was an implementation study [39]. Trial-based costing studies are conducted under ideal conditions and, hence have low external validity. Treatments provided to the children also varied between studies depending on the country’s treatment policy, drug availability, type of severe malaria, and the associated co-morbidities; all of these involve the use of different combinations of drugs. The distinction between severe malaria and severe malaria anaemia was not always clear as some studies reported that patients with severe malaria received blood transfusion – a standard treatment of severe anaemia while others did not disaggregate the costs or mixed different types of malaria and instead labelled the group as having severe malaria [42, 45, 46, 50, 51]. The length of hospital stays also impacts the cost of treatment, however only nine studies reported the number of admission days. Three studies reported three or fewer days [39, 46, 52, 55], and in five studies, the number of days varied between 4 and 9.3 days [37, 38, 42, 50, 51].

Another source of heterogeneity was the study settings (urban, rural), ownership of facilities (public, faith-based/mission, university or private), and the levels of health facilities depending on the health system organization (health centres, district, sub-district, county, provincial, and regional hospitals). Practically, it is impossible to eliminate some of these variances in future studies because researchers adapt their methodologies to the purpose of study, data availability, and simplification of data collection, analysis, and reporting. Therefore, these factors must be considered when making comparisons between countries of the costs of treating malaria. Nonetheless, providing a more detailed cost breakdown within cost studies would facilitate a more accurate comparison of studies.

Like the provider costs, household costs varied between studies mainly due to cost items or categories included. Household costs can be direct medical costs such as fees paid at the facilities for registration, consultations, medicines, or hospitalizations; direct non-medical such as transportation, food, and drinks; and indirect costs like the loss in productivity due to time spent seeking care or taking care of a sick child. Some studies for example by Mbalabu and Moukoumbi et al. in DR Congo and Gabon only included the direct medical costs [53, 54] while the study by Abotsi et al. in Ghana (2012) included transport and indirect costs [45]. Studies by Watts (Kenya) [52], Hennessee (Malawi) [43] and Onwujekwe (Nigeria) [40] included direct medical and non-medical costs and indirect costs. The study by Ilunga-Ilunga et al. in Congo DR, which had the second highest cost per patient, included pre-hospital costs incurred on self-medication, health center or church attendance, and traditional therapy [44]. Similarly, the study by Alonso et al. [42] in Mozambique, which had the third largest household costs included self-treatment costs and the costs of seeking health care from traditional healers.

When household expenditures were compared to the monthly GNI per capita as a proxy of household income all but one exceeded the 10% threshold; hence, can be categorized as catastrophic health expenditure (CHE). In all countries except Kenya and Nigeria, the reported household costs were more than twice the threshold income. In Zambia, household costs related to the treatment of severe malaria in children have been estimated to represent about 26% of the mean monthly income [6]. In DR Congo, the incidence of CHE due to severe malaria in children was estimated at 46% [12], while in Sudan, South Africa, Mozambique, and Zimbabwe the incidence varied between 17 and 32% [13–15].

This study has several limitations: First, the existing studies are very heterogeneous in terms of the methodology used, the types of resources included, facility levels, ownership, and design, thus making any comparison difficult. Second, the range and median values reported need to be interpreted with care considering the heterogeneity of the studies included. Lastly, monthly GNI per capita was used as a proxy for individual household income. This may have overestimated or underestimated the degree of CHE because the use of GNI assumes that all households have the same income, which is not the case, not least in developing countries.

### Policy implication

This study shows that severe malaria in children imposes a significant economic burden on households as expressed by the degree of CHE. Therefore, policies that protect households from impoverishing severe malaria treatment costs need to be enacted if these countries are to realize universal health coverage by 2030, as targeted in the Sustainable Development Goals [62]. In addition, the study shows that most of the existing cost data is older than ten years. Therefore, this study recommends new costing studies that will reflect the new realities of malaria control initiatives including the decreasing malaria transmission and the shift of disease burden to older children.

## Conclusion

Evidence about the cost of managing severe malaria in children in Africa is relatively scarce. Despite the stated limitations, the existing studies show that the disease imposes a significant economic burden on the health system and households. This study calls for more standardized costing studies to fill the existing gap, particularly in high-burden high-impact countries, to inform resource allocation decisions.

## Supplementary Information

Supplementary Material 1

## Data Availability

No datasets were generated or analysed during the current study.

## Abbreviations

CHE: Catastrophic health expenditure
WHO: World Health Organization
CCEMG: Campbell and Cochrane Economics Methods Group
EPPI: Evidence for Policy and Practice Information
GNI: Gross National Income
ABC: Activity-Based Costing
TDABC: Time-Driven Activity-Based Costing
PDMC: Post-discharge malaria chemoprevention

## References

[1] WHO. World malaria report 2023. Geneva: World Health Organization; 2023.

[2] WHO: High burden to high impact: a targeted malaria response. https://www.who.int/publications/i/item/WHO-CDS-GMP-2018.25. Geneva: World Health Organization; 2018.

[3] Sachs J, Malaney P. The economic and social burden of malaria. Nature. 2002;415:680–5. 11832956 10.1038/415680a

[4] White MT, Conteh L, Cibulskis R, Ghani AC. Costs and cost-effectiveness of malaria control interventions—a systematic review. Malar J. 2011;10:337. 22050911 10.1186/1475-2875-10-337 PMC3229472

[5] Sicuri E, Vieta A, Lindner L, Constenla D, Sauboin C. The economic costs of malaria in children in three sub-Saharan countries: Ghana, Tanzania and Kenya. Malar J. 2013;12:307. 24004482 10.1186/1475-2875-12-307 PMC3844618

[6] Mtalimanja M, Abasse KS, Mtalimanja JL, Yuan XZ, Wenwen D, Xu W. Economic evaluation of severe malaria in children under 14 years in Zambia. Cost Eff Resour Alloc. 2022;20:4. 35123482 10.1186/s12962-022-00340-9 PMC8817518

[7] Singh MP, Saha KB, Chand SK, Sabin LL. The economic cost of malaria at the household level in high and low transmission areas of central India. Acta Trop. 2019;190:344–9. 30521804 10.1016/j.actatropica.2018.12.003

[8] Somi MF, Butler JR, Vahid F, Njau JD, Kachur SP, Abdulla S. Economic burden of malaria in rural Tanzania: variations by socioeconomic status and season. Trop Med Int Health. 2007;12:1139–47. 17956495 10.1111/j.1365-3156.2007.01899.x

[9] Chuma JM, Thiede M, Molyneux CS. Rethinking the economic costs of malaria at the household level: evidence from applying a new analytical framework in rural Kenya. Malar J. 2006;5:76. 16939658 10.1186/1475-2875-5-76 PMC1570360

[10] Xu K: Distribution of payments and catastrophic expenditures methodogy. Meeting Report. [Internet]. Geneva: World Health Organization; 2004. https://www.who.int/publications/i/item/EIP-FER-DP.05.2

[11] Wagstaff A, van Doorslaer E. Catastrophe and impoverishment in paying for health care: with applications to Vietnam 1993–1998. Health Econ. 2003;12:921–34. 14601155 10.1002/hec.776

[12] Ilunga-Ilunga F, Levêque A, Laokri S, Dramaix M. Incidence of catastrophic health expenditures for households: an example of medical attention for the treatment of severe childhood malaria in Kinshasa reference hospitals, Democratic Republic of Congo. J Infect Public Health. 2015;8:136–44. 25264234 10.1016/j.jiph.2014.08.008

[13] Castillo-Riquelme M, McIntyre D, Barnes K. Household burden of malaria in South Africa and Mozambique: is there a catastrophic impact? Trop Med Int Health. 2008;13:108–22. 18291009 10.1111/j.1365-3156.2007.01979.x

[14] Abdel-Hameed AA, Abdalla HM, Alnaury AH. Household expenditure on malaria case management in Wad-Medani. Sudan Afr J Med Med Sci. 2001;30(Suppl):35–8. 14513936

[15] Gunda R, Shamu S, Chimbari MJ, Mukaratirwa S. Economic burden of malaria on rural households in Gwanda district, Zimbabwe. Afr J Prim Health Care Fam Med. 2017;9:e1–6. 28893077 10.4102/phcfm.v9i1.1317 PMC5594239

[16] Chima RI, Goodman CA, Mills A. The economic impact of malaria in Africa: a critical review of the evidence. Health Policy. 2003;63:17–36. 12468115 10.1016/s0168-8510(02)00036-2

[17] El-Houderi A, Constantin J, Castelnuovo E, Sauboin C. Economic and resource use associated with management of malaria in children aged <5 years in Sub-Saharan Africa: a systematic literature review. MDM Policy Pract. 2019;4:2381468319893986. 31903421 10.1177/2381468319893986 PMC6927205

[18] Conteh L, Shuford K, Agboraw E, Kont M, Kolaczinski J, Patouillard E. Costs and cost-effectiveness of malaria control interventions: a systematic literature review. Value Health. 2021;24:1213–22. 34372987 10.1016/j.jval.2021.01.013 PMC8324482

[19] Andrade MV, Noronha K, Diniz BPC, Guedes G, Carvalho LR, Silva VA, et al. The economic burden of malaria: a systematic review. Malar J. 2022;21:283. 36199078 10.1186/s12936-022-04303-6 PMC9533489

[20] Okell LC, Kwambai TK, Dhabangi A, Khairallah C, Nkosi-Gondwe T, Winskill P, et al. Projected health impact of post-discharge malaria chemoprevention among children with severe malarial anaemia in Africa. Nat Comm. 2023;14:402. 10.1038/s41467-023-35939-w PMC9876927 36697413

[21] WHO: Guidelines for malaria, 14 March 2023. (WHO/UCN/GMP/2023.01)**.** Geneva: World Health Organization; 2023.

[22] Snow RW, Omumbo JA, Lowe B, Molyneux CS, Obiero JO, Palmer A, et al. Relation between severe malaria morbidity in children and level of *Plasmodium falciparum* transmission in Africa. Lancet. 1997;349:1650–4. 9186382 10.1016/S0140-6736(97)02038-2

[23] Plewes K, Leopold SJ, Kingston HWF, Dondorp AM. Malaria: What’s New in the Management of Malaria? Infect Dis Clin North Am. 2019;33:39–60. 30712767 10.1016/j.idc.2018.10.002

[24] Coulibaly D, Kone AK, Kane B, Guindo B, Tangara B, Sissoko M, et al. Shifts in the clinical epidemiology of severe malaria after scaling up control strategies in Mali. Front Neurol. 2022;13: 988960. 36523346 10.3389/fneur.2022.988960 PMC9744791

[25] Page MJ, McKenzie JE, Bossuyt PM, Boutron I, Hoffmann TC, Mulrow CD, et al. The PRISMA 2020 statement: an updated guideline for reporting systematic reviews. BMJ. 2021;372: n71. 33782057 10.1136/bmj.n71 PMC8005924

[26] Mori AT, Mallange GJ: Cost of managing severe malaria in children in malaria endemic countries in africa: a systematic review. PROSPERO 2023 CRD42023401799. 2023. https://www.crdyorkacuk/prospero/display_recordphp?ID=CRD42023401799.

[27] Kwambai TK, Mori AT, Nevitt S, van Eijk AM, Samuels AM, Robberstad B, et al. Post-discharge morbidity and mortality in children admitted with severe anaemia and other health conditions in malaria-endemic settings in Africa: a systematic review and meta-analysis. Lancet Child Adolesc Health. 2022;6:474–83. 35605629 10.1016/S2352-4642(22)00074-8 PMC10196725

[28] Creese A, Parker D: Cost analysis in primary health care: A training manual for programme managers. [Internet]. Geneva: World Health Organization; 1994. https://iris.who.int/handle/10665/40030

[29] McIntyre D, Thiede M, Dahlgren G, Whitehead M. What are the economic consequences for households of illness and of paying for health care in low- and middle-income country contexts? Soc Sci Med. 2006;62:858–65. 16099574 10.1016/j.socscimed.2005.07.001

[30] Drummond MF, Sculpher MJ, Torrance GW, O’Brien BJ, Stoddart GL. Methods for the economic evaluation of health care programmes. 3rd ed. New York: Oxford University Press; 2005.

[31] van Lier LI, Bosmans JE, van Hout HPJ, Mokkink LB, van den Hout WB, de Wit GA, et al. Consensus-based cross-European recommendations for the identification, measurement and valuation of costs in health economic evaluations: a European Delphi study. Eur J Health Econ. 2018;19:993–1008. 29260341 10.1007/s10198-017-0947-x PMC6105226

[32] Husereau D, Drummond M, Petrou S, Carswell C, Moher D, Greenberg D, et al. Consolidated health economic evaluation reporting standards (CHEERS) statement. Cost Eff Resour Alloc. 2013;11:6. 23531194 10.1186/1478-7547-11-6 PMC3607888

[33] Schnitzler L, Roberts TE, Jackson LJ, Paulus ATG, Evers SMAA. A consensus-based checklist for the critical appraisal of cost-of-illness (COI) studies. Int J Technol Assess Health Care. 2023;39: e34. 37325977 10.1017/S0266462323000193 PMC11574538

[34] Shemilt I, James T, Marcello M. A web-based tool for adjusting costs to a specific target currency and price year. Evid Policy. 2010;6:51–9.

[35] World Bank. https://data.worldbank.org/indicator/NY.GNP.PCAP.CD

[36] Mandrik O, Severens JL, Bardach A, Ghabri S, Hamel C, Mathes T, et al. Critical appraisal of systematic reviews with costs and cost-effectiveness outcomes: an ISPOR good practices task force report. Value Health. 2021;24:463–72. 33840423 10.1016/j.jval.2021.01.002

[37] Ayieko P, Akumu AO, Griffiths UK, English M. The economic burden of inpatient paediatric care in Kenya: Household and provider costs for treatment of pneumonia, malaria and meningitis. Cost Effd Resour Alloc. 2009;7:3. 10.1186/1478-7547-7-3 PMC2640355 19161598

[38] Comfort AB, Van Dijk JH, Thuma PE, Mharakurwa S, Gabert R, Korde S, et al. Hospitalizations and costs incurred at the facility level after scale-up of malaria control: pre-post comparisons from two hospitals in Zambia. Am J Trop Med Hyg. 2014;90:20–32. 24218409 10.4269/ajtmh.13-0019 PMC3886421

[39] Ferrari G, Ntuku HM, Burri C, Tshefu AK, Duparc S, Hugo P, et al. An operational comparative study of quinine and artesunate for the treatment of severe malaria in hospitals and health centres in the Democratic Republic of Congo: the MATIAS study. Malar J. 2015;14:226. 26024661 10.1186/s12936-015-0732-1 PMC4455055

[40] Onwujekwe O, Uguru N, Etiaba E, Chikezie I, Uzochukwu B, Adjagba A. The economic burden of malaria on households and the health system in Enugu State southeast Nigeria. PLoS ONE. 2013;8: e78362. 24223796 10.1371/journal.pone.0078362 PMC3817251

[41] Lubell Y, Riewpaiboon A, Dondorp AM, von Seidlein L, Mokuolu OA, Nansumba M, et al. Cost-effectiveness of parenteral artesunate for treating children with severe malaria in sub-Saharan Africa. Bull World Health Organ. 2011;89:504–12. 21734764 10.2471/BLT.11.085878 PMC3127273

[42] Alonso S, Chaccour CJ, Elobolobo E, Nacima A, Candrinho B, Saifodine A, et al. The economic burden of malaria on households and the health system in a high transmission district of Mozambique. Malar J. 2019;18:360. 31711489 10.1186/s12936-019-2995-4 PMC6849240

[43] Hennessee I, Chinkhumba J, Briggs-Hagen M, Bauleni A, Shah MP, Chalira A, et al. Household costs among patients hospitalized with malaria: evidence from a national survey in Malawi, 2012. Malar J. 2017;16:395. 28969643 10.1186/s12936-017-2038-y PMC5625606

[44] Ilunga-Ilunga F, Leveque A, Ngongo LO, Kandolo FT, Dramaix M. Costs of treatment of children affected by severe malaria in reference hospitals of Kinshasa, democratic Republic of Congo. J Infect Dev Ctries. 2014;8:1574–83. 25500655 10.3855/jidc.4622

[45] Abotsi AK. Cost burden of infant malaria treatment on households and health institutions in the Upper East Region. Univ Cape Coast J Arts Soc Sci. 2012;1:168–89.

[46] Maka DE, Chiabi A, Obadeyi B, Mah E, Nguefack S, Nana P, et al. Economic evaluation of artesunate and three quinine regimens in the treatment of severe malaria in children at the Ebolowa Regional Hospital-Cameroon: a cost analysis. Malar J. 2016;15:587. 27923381 10.1186/s12936-016-1639-1 PMC5142138

[47] Mori AT, Ngalesoni F, Norheim OF, Robberstad B. Cost-effectiveness of dihydroartemisinin-piperaquine compared with artemether-lumefantrine for treating uncomplicated malaria in children at a district hospital in Tanzania. Malar J. 2014;13:363. 25223864 10.1186/1475-2875-13-363 PMC4171550

[48] Batura N, Kasteng F, Condoane J, Bagorogosa B, Castel-Branco AC, Kertho E, et al. Costs of treating childhood malaria, diarrhoea and pneumonia in rural Mozambique and Uganda. Malar J. 2022;21:239. 35987625 10.1186/s12936-022-04254-y PMC9392282

[49] Kirigia JM, Snow RW, Fox-Rushby J, Mills A. The cost of treating paediatric malaria admissions and the potential impact of insecticide-treated mosquito nets on hospital expenditure. Trop Med Int Health. 1998;3:145–50. 9537277 10.1046/j.1365-3156.1998.00204.x

[50] Koné I, Marschall P, Flessa S. Costing of Malaria treatment in a rural district hospital. Health. 2010;2:759–68.

[51] Kühl MJ, Gondwe T, Dhabangi A, Kwambai TK, Mori AT, Opoka R, et al. Economic evaluation of postdischarge malaria chemoprevention in preschool children treated for severe anaemia in Malawi, Kenya, and Uganda: a cost-effectiveness analysis. EClinicalMedicine. 2022;52: 101669. 36313146 10.1016/j.eclinm.2022.101669 PMC9596312

[52] Watts C, Atieli H, Alacapa J, Lee M-C, Zhou G, Githeko A, Yan G, Wiseman V. Rethinking the economic costs of hospitalization for malaria: accounting for the comorbidities of malaria patients in western Kenya. Malar J. 2021;20:429. 34717637 10.1186/s12936-021-03958-x PMC8557520

[53] Mbalabu OB, Tshibangu EK, Disashi GT, Mantshumba J-C, Bobanga T. Direct cost of management and clinical profile of severe malaria in Paediatric Hospital of Mbujimayi. Congo: Research Square; 2021.

[54] Moukoumbi LG, Ngoungou EB, Ibinga E, Engohang-Ndong J, Wittwer J. Evaluation of direct costs associated with the management of clinical stage of malaria in children under five years old in Gabon. Malar J. 2021;20:334. 34330288 10.1186/s12936-021-03862-4 PMC8325277

[55] Kodhiambo MO, Oyugi JO, Amugune BK. Modelling the household cost of paediatric malaria treatment in a rural county in Kenya: do non-user fee payments matter? A partial cost of illness analysis. BMJ Open. 2020;10: e033192. 32205372 10.1136/bmjopen-2019-033192 PMC7103840

[56] WHO recommends grounbreaking malaria vaccine for children at risk. https://www.who.int/news/item/06-10-2021-who-recommends-groundbreaking-malaria-vaccine-for-children-at-risk

[57] Špacírová Z, Epstein D, García-Mochón L, Rovira J. A general framework for classifying costing methods for economic evaluation of health care. Eur J Health Econ. 2020;21:529–42. 31960181 10.1007/s10198-019-01157-9 PMC8149350

[58] Cunnama L, Sinanovic E, Ramma L, Foster N, Berrie L, Stevens W, Molapo S, Marokane P, McCarthy K, Churchyard G, Vassall A. Using top-down and bottom-up costing approaches in LMICs: the case for using both to assess the incremental costs of new technologies at scale. Health Econ. 2016;25:53–66. 26763594 10.1002/hec.3295 PMC5066665

[59] da Silva Etges APB, Cruz LN, Notti RK, Neyeloff JL, Schlatter RP, Astigarraga CC, et al. An 8-step framework for implementing time-driven activity-based costing in healthcare studies. Eur J Health Econ. 2019;20:1133–45. 31286291 10.1007/s10198-019-01085-8

[60] Kaplan RS. Time-driven activity-based costing: a simpler and more powerful path to higher profits. Boston: Harvard Business School Press; 2007.

[61] Chapko MK, Liu C-F, Perkins M, Li Y-F, Fortney JC, Maciejewski ML. Equivalence of two healthcare costing methods: bottom-up and top-down. Health Econ. 2009;18:1188–201. 19097041 10.1002/hec.1422

[62] UNGA: Seventieth session United Nations General Assembly-Transforming our world: the 2030 Agenda for Sustainable Development (A/RES/70/1). New York: United Nations; 2015.

