# Supplementary material for: Cost of treating severe malaria in children in Africa: a systematic literature review
Source: Malar J. 2024 Nov 9;23:334. doi: 10.1186/s12936-024-05173-w (PMC11550527; doi:10.1186/s12936-024-05173-w)
Supplement: Supplementary file 1 — Supplementary Material 1 [file 12936_2024_5173_MOESM1_ESM.docx]

**Additional file 1.**

Ovid MEDLINE(R) and Epub Ahead of Print, In-Process, In-Data-Review & Other Non-Indexed Citations and Daily <1946 to January 04, 2024>

1. (severe malaria or cerebral malaria or severe malaria anaemia or severe malaria anemia or hospital malaria or inpatient malaria).ti,ab,kf.

2. (cost benefit or cost-benefit or cost effectiveness or cost-effectiveness or cost utility or cost analy* or cost and benefit or economic evaluation* or cost or economic benefit*).ti,ab,kf.

3. ("Burkina Faso" or Burkinabe or Cameroon* or "Democratic Republic of the Congo" or Congolese or Ghana* or Mali* or Mozambique or Mozambican or Niger* or Tanzania* or Uganda* or Angola* or Benin or Beninese or Botswana or Motswana or Burundi* or "Central African Republic" or Chad* or Comoros or Comorian* or "Cote d'Ivoire" or "Ivory Coast*" or Djibouti* or "Equatorial Guinea*" or Eritrea* or Ethiopia* or Gabon* or Gambia* or Guinea* or Guinea-Bissau or Bissau-Guinean* or Kenya* or Liberia* or Madagascar or Malagasy or Malawi* or Maurit* or Namibia* or "Republic of Congo" or Congolese or Rwand* or Senegal* or "Sierra Leone" or Somalia* or "South Africa" or "South African" or "South Sudan" or Sudan* or Togo* or Zambia* or Zimbabwe*).ti,ab,kf.

4. 1 and 2 and 3

**Embase** <1974 to 2024 January 04>

1. (severe malaria or cerebral malaria or severe malaria anaemia or severe malaria anemia or hospital malaria or inpatient malaria).ti,ab,kf.

2. (((cost benefit or cost-benefit or cost effectiveness or cost-effectiveness or cost utility or cost analy* or cost and benefit or economic evaluation* or cost or economic benefit*).ti,ab,kf.

3. ("Burkina Faso" or Burkinabe or Cameroon* or "Democratic Republic of the Congo" or Congolese or Ghana* or Mali* or Mozambique or Mozambican or Niger* or Tanzania* or Uganda* or Angola* or Benin or Beninese or Botswana or Motswana or Burundi* or "Central African Republic" or Chad* or Comoros or Comorian* or "Cote d'Ivoire" or "Ivory Coast*" or Djibouti* or "Equatorial Guinea*" or Eritrea* or Ethiopia* or Gabon* or Gambia* or Guinea* or Guinea-Bissau or Bissau-Guinean* or Kenya* or Liberia* or Madagascar or Malagasy or Malawi* or Maurit* or Namibia* or Rwand* or Senegal* or "Sierra Leone" or Somalia* or "South Africa*" or "South African" or "South Sudan*" or Sudan* or Togo* or Zambia* or Zimbabwe*).ti,ab,kf.

4. 1 and 2 and 3 81

**WEB OF SCIENCE**

1. ((((((TS=("severe malaria")) OR TS=("cerebral malaria")) OR TS=("severe malaria anaemia")) OR TS=("severe malaria anemia")) OR TS=("hospital malaria")) OR TS=("inpatient malaria").TI,AB,AK.)
2. (((((((((TS=("cost benefit" )) OR TS=("cost-benefit")) OR TS=("cost effectiveness")) OR TS=("cost-effectiveness")) OR TS=("cost utility")) OR TS=("cost analy*")) OR TS=("economic evaluation*" )) OR TS=(cost)) OR TS=("economic benefit*").TI,AB,AK.)
3. ((((((((((((((((((((((((((((((((((((((((((((((((((TS=("Burkina Faso" )) OR TS=(Burkinabe)) OR TS=(Cameroon*)) OR TS=("Democratic Republic of the Congo")) OR TS=(Congolese)) OR TS=(Ghana*)) OR TS=(Mali*)) OR TS=(Mozambique)) OR TS=(Mozambican)) OR TS=(Niger*)) OR TS=(Tanzania*)) OR TS=(Uganda*)) OR TS=(Angola*)) OR TS=(Benin)) OR TS=(Beninese)) OR TS=(Botswana)) OR TS=(Motswana)) OR TS=(Burundi*)) OR TS=("Central African Republic" )) OR TS=(Chad*)) OR TS=(Comoros)) OR TS=(Comorian*)) OR TS=("Cote d'Ivoire")) OR TS=("Ivory Coast*" )) OR TS=(Djibouti*)) OR TS=("Equatorial Guinea*" )) OR TS=(Eritrea*)) OR TS=(Ethiopia* )) OR TS=(Gabon*)) OR TS=(Gambia* )) OR TS=(Guinea*)) OR TS=("Guinea-Bissau")) OR TS=("Bissau-Guinean*")) OR TS=(Kenya*)) OR TS=(Liberia*)) OR TS=(Madagascar)) OR TS=(Malagasy)) OR TS=(Malawi* )) OR TS=(Maurit*)) OR TS=(Namibia*)) OR TS=(Rwand*)) OR TS=(Senegal*)) OR TS=("Sierra Leone" )) OR TS=(Somalia* )) OR TS=("South Africa*" )) OR TS=("South Sudan*" )) OR TS=(Sudan* )) OR TS=(Togo*)) OR TS=(Zambia*)) OR TS=(Zimbabwe*).TI,AB,AK.)
4. #1 AND #2 AND #3

**CINAHIL**

| **#** | **Query** |  | **Last Run Via** |
| --- | --- | --- | --- |
| S4 | S1 AND S2 AND S3 |  | Interface - EBSCOhost Research Databases Search Screen - Advanced Search Database - CINAHL |
| S3 | TX "Burkina Faso" or Burkinabe or Cameroon* or "Democratic Republic of the Congo" or Congolese or Ghana* or Mali* or Mozambique or Mozambican or Niger* or Tanzania* or Uganda* or Angola* or Benin or Beninese or Botswana or Motswana or Burundi* or "Central African Republic" or Chad* or Comoros or Comorian* or "Cote d'Ivoire" or "Ivory Coast*" or Djibouti* or "Equatorial Guinea*" or Eritrea* or Ethiopia* or Gabon* or Gambia* or Guinea* or Guinea-Bissau or Bissau-Guinean* or Kenya* or Liberia* or Madagascar or Malagasy or Malawi* or Maurit* or Namibia* or "Republic of Congo" or Congolese or Rwand* or Senegal* or "Sierra Leone" or Somalia* or "South Africa" or "South African" or "South Sudan" or Sudan* or Togo* or Zambia* or Zimbabwe* |  | Interface - EBSCOhost Research Databases Search Screen - Advanced Search Database - CINAHL |
| S2 | TX cost benefit or cost-benefit or cost effectiveness or cost-effectiveness or cost utility or cost analy* or cost and benefit or economic evaluation* or cost or economic benefit* |  | Interface - EBSCOhost Research Databases Search Screen - Advanced Search Database - CINAHL |
| S1 | TX severe malaria or cerebral malaria or severe malaria anaemia or severe malaria anemia or hospital malaria or inpatient malaria |  | Interface - EBSCOhost Research Databases Search Screen - Advanced Search Database - CINAHL |
